# Hospital Episode Statistics data analysis of postoperative venous thromboembolus in patients undergoing urological surgery: a review of 126,891 cases

**DOI:** 10.1308/003588413X13511609956219

**Published:** 2013-01

**Authors:** J Dyer, S Wyke, C Lynch

**Affiliations:** ^1^Royal Liverpool and Broadgreen University Hospitals NHS Trust,UK; ^2^Liverpool John Moores University,UK

**Keywords:** Urology, Venous thromboembolism, Pulmonary embolism, Deep vein thrombosis

## Abstract

**Introduction:**

Current guidelines on venous thromboembolism (VTE) prevention do not reflect the potential varying risk for patients undergoing different urological procedures. Our study aimed to establish the procedure specific rate of postoperative VTE in patients undergoing urological surgery.

**Methods:**

Hospital Episode Statistics were obtained for all patients undergoing common urological procedures between April 2009 and April 2010. This cohort was followed up to identify all patients reattending with either deep vein thrombosis (DVT) or pulmonary embolism (PE) within 12 months.

**Results:**

A total of 126,891 individuals underwent urological surgery during the study period. This included 89,628 men (70.6%) and 37,236 women (29.3%) with a mean age of 65.2 years. At the 12-month follow-up, 839 patients (0.66%) were readmitted with VTE. Of these, 373 (0.29%) were admitted with DVT and 466 (0.37%) with PE. The procedure-specific rate of VTE varied significantly between 2.86% following cystectomy and 0.23% following urethral dilatation. Procedures performed in the lithotomy position carried a significantly lower risk of VTE than those performed in the supine position (0.60% vs 1.28%, *p*<0.0001). Furthermore, of all procedures performed in the lithotomy position, those performed on benign conditions carried a significantly lower risk than those performed on malignant disease (0.52% vs 0.79%, *p*<0.0001).

**Conclusions:**

Procedure specific rates of postoperative VTE vary widely among patients undergoing urological procedures. These findings suggest the potential benefit of prolonging the use of thromboprophylaxis in high-risk patients but also exploring the apparent lack of need for routine thromboprophylaxis in patients undergoing low-risk procedures.

The House of Commons Health Committee 2005 report on venous thromboembolism (VTE) documents the significant problems associated with hospital-acquired VTE.^1^ It estimates the annual incidence of fatal hospital-acquired pulmonary embolism (PE) at 25,000 annually and highlights the substantial morbidity associated with deep vein thrombosis (DVT). Consequently, in 2010 the National Institute for Health and Clinical Excellence (NICE) published guidance for the care of hospitalised patients at risk of developing VTE.^2^ These guidelines detail the suggested mechanical, medical and surgical measures that should be employed in hospitalised patients suffering with medical illness and undergoing a range of surgical procedures.

Clearly, both the risk of VTE and the consequence of postoperative bleeding differ between different surgical procedures. This is reflected in the NICE guidance with different recommendations for surgery in different specialties (orthopaedic, ophthalmology, gastrointestinal etc).^2^ There is, however, no procedure-specific variation in guidance for patients undergoing urological surgery. This is particularly important in urological surgery as there is wide variation in surgical techniques (open, endoscopic and laparoscopic), in patient positioning (supine, prone and lithotomy) and in diseases (benign and malignant). Furthermore, much of the data on which conclusions for current guidelines are based date from the 1970s and 1980s.^3–7^ By interrogating the National Health Service (NHS) Hospital Episode Statistics (HES) database, our study aimed to elucidate the rate of procedure-specific postoperative VTE in patients undergoing a range of urological surgery.

## Methods

HES data were obtained for all patients undergoing common urological procedures in NHS trusts throughout England between April 2009 and April 2010. The codes and groupings are given in Table 1. This dataset was employed as it was the most recently available complete dataset that permitted a minimum follow-up period of 12 months. The cohort was followed to identify those patients reattending with either DVT or PE within 12 months of their surgery. Patients were stratified according to sex, procedure and operative position. Statistical significance was determined using a chi-squared test. In order to preserve patient confidentiality, data were not included in subsequent analysis if the output from HES was fewer than six in any given category.

**Table 1:** Hospital Episode Statistics codes for commonly performed urological procedures

**Nephrectomy**
M02.1 Nephrectomy and excision of perirenal tissue M02.2 Nephroureterectomy NEC M02.3 Bilateral nephrectomy M02.4 Excision of half of horseshoe kidney M02.5 Nephrectomy NEC M02.6 Excision of rejected transplanted kidney M02.8 Other specified total excision of kidney M02.9 Unspecified total excision of kidney
**Endoscopic ureteric or renal calculus procedures**
M09.1 Endoscopic ultrasonography fragmentation of calculus of kidney M09.2 Endoscopic electrohydraulic shock wave fragmentation of calculus of kidney M09.3 Endoscopic laser fragmentation of calculus of kidney M09.4 Endoscopic extraction of calculus of kidney NEC M09.8 Other specified therapeutic endoscopic operations on calculus of kidney M09.9 Unspecified therapeutic endoscopic operations on calculus of kidney M11.3 Diagnostic endoscopic retrograde examination of kidney NEC M27.1 Ureteroscopic laser fragmentation of calculus of ureter M27.2 Ureteroscopic fragmentation of calculus of ureter NEC M27.3 Ureteroscopic extraction of calculus of ureter M27.4 Ureteroscopic insertion of ureteric stent M27.5 Ureteroscopic removal of ureteric stent M28.1 Endoscopic laser fragmentation of calculus of ureter NEC M28.2 Endoscopic fragmentation of calculus of ureter NEC M28.3 Endoscopic extraction of calculus of ureter NEC M28.4 Endoscopic catheter drainage of calculus of ureter M28.5 Endoscopic drainage of calculus of ureter by dilation of ureter M28.8 Other endoscopic removal of calculus from ureter, other specified M28.9 Unspecified other endoscopic removal of calculus from ureter M29.2 Endoscopic insertion of tubal prosthesis into ureter NEC M29.5 Endoscopic renewal of tubal prosthesis into ureter M30.1 Endoscopic retrograde pyelography M30.9 Unspecified diagnostic endoscopic examination of ureter
**Cystectomy**
M34.1 Cystoprostatectomy M34.2 Cystourethrectomy M34.3 Cystectomy NEC M34.4 Simple cystectomy M34.8 Other specified total excision of bladder M34.9 Unspecified total excision of bladder
**prostatectomy**
M61.1 Total excision of prostate and capsule of prostate
**TURBT/cystoscopic bladder procedures (malignant)**
M42.1 Endoscopic resection of lesion of bladder M42.2 Endoscopic cauterisation of lesion of bladder M42.3 Endoscopic destruction of lesion of bladder NEC M42.8 Other specified endoscopic extirpation of lesion of bladder M42.9 Unspecified endoscopic extirpation of lesion of bladder
**Cystoscopic bladder procedures (benign)**
M43.2 Endoscopic hydrostatic distension of bladder M44.1 Endoscopic lithopaxy M44.2 Endoscopic extraction of calculus of bladder NEC M44.3 Endoscopic removal of foreign body from bladder M44.4 Endoscopic removal of blood clot from bladder M44.8 Other therapeutic endoscopic operations on bladder, other specified M44.9 Unspecified other therapeutic endoscopic operations on bladder M45.3 Diagnostic endoscopic examination of bladder and biopsy of lesion of bladder using rigid cystoscope M45.4 Diagnostic endoscopic examination of bladder and biopsy of lesion of prostate using rigid cystoscope M45.5 Diagnostic endoscopic examination of bladder using rigid cystoscope
**TURP/bladder outflow surgery**
M65.1 Endoscopic resection of prostate using electrotome M65.3 Endoscopic resection of prostate NEC M65.4 Endoscopic resection of prostate using laser M66.2 Endoscopic incision of outlet of male bladder NEC
**Urethral procedures**
M76.4 Endoscopic dilation of urethra M79.2 Dilation of urethra NEC

NEC = not elsewhere classified; TURBT = transurethral resection of bladder tumour; TURP = transurethral resection of prostate

**Figure 1:**
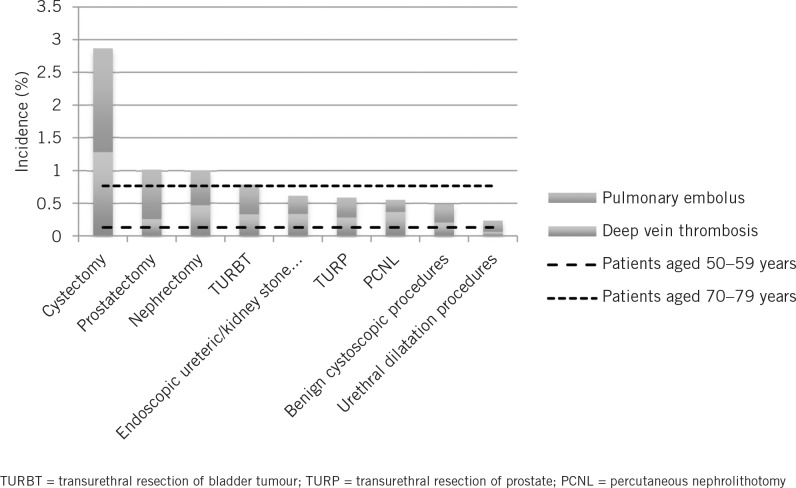
Procedure-specific variation in postoperative venous thromboembolism (VTE) including representations of community-based incidence of VTE taken from White^9^

## Results

A total of 126,891 individuals were identified as having undergone urological surgery during the study period. This included 89,628 men (70.6%), 37,236 women (29.3%) and 27 patients with sex unknown (0.02%). The mean patient age was 65.2 years. At the 12-month follow-up, 839 patients (0.66%) were readmitted with VTE. Of these, 373 (0.29%) had been admitted with DVT and 466 (0.37%) with PE. There was no significant difference between the incidence of VTE between men and women (0.67% vs 0.63%, *p*=0.413).

Procedure-specific VTE rates varied considerably (Fig 1, Table 2) with major open and laparoscopic surgery carrying the highest risk of VTE. Of this group, cystectomy carried the highest risk with an incidence of 2.86%. Benign cystoscopic procedures and urethral dilatation carried the lowest risk of postoperative VTE with 0.48% and 0.23% respectively. Procedures performed in the lithotomy position were associated with approximately half the risk of developing VTE than those performed in the supine position (0.60% vs 1.28%, *p*<0.0001) (Table 3). Furthermore, there was a reduction in VTE risk when comparing cystoscopic surgery on benign and malignant conditions (0.52% vs 0.79%, *p*<0.0001) (Table 4).

## Discussion

As expected, procedure-specific rates of symptomatic VTE varied greatly among patients undergoing urological surgery. Cystectomy appears to carry the highest risk of VTE, which is greater than the published incidence for known high-risk procedures such as total hip arthroplasty (2.4%), partial hip arthroplasty (2.0%) and embolectomy of lower limb artery (2.8%).^8^

Current guidelines for major urological, gastrointestinal and gynaecological pelvic cancer surgery suggest continuing medical thromboprophylaxis following discharge for 28 days.^2^ Although the assumption from our findings would be to support a prolonged duration of postoperative thromboprophylaxis, it is necessary to reiterate that our study indicates annual incidence of VTE rather than the incidence in a different specific time period. There is a need for further clarification on the VTE risk for such patients, particularly in terms of the time to development of symptomatic VTE. Such knowledge will aid the development of recommendations for patients undergoing a cystectomy with regard to method, duration and dosage of thromboprophylaxis. Conversely, our study showed that urethral dilatation and cystoscopic procedures performed in the lithotomy position for benign conditions (eg removal of bladder clot, cystolithopaxy, removal of foreign body and cystodistension) carry a very low risk of VTE (0.23–0.48%).

**Table 2:** Venous thromboembolism, deep vein thrombosis and pulmonary embolism incidence in commonly performed urological procedures

Procedure	Total procedures	VTE		DVT		PE		Mean age of onset of VTE
		*n*	Incidence	*n*	Incidence	*n*	Incidence	
Cystectomy	1,641	47	2.86%	21	1.28%	26	1.58%	66.1 years
Prostatectomy	3,213	32	1.00%	15	0.47%	17	0.53%	72.5 years
Nephrectomy	6,230	63	1.01%	16	0.26%	47	0.75%	62.2 years
TURBT	35,765	281	0.79%	118	0.33%	163	0.46%	55.2 years
Ureteric/kidney stone removal	27,133	166	0.61%	91	0.34%	75	0.28%	70.5 years
TURP	25,691	150	0.58%	72	0.28%	78	0.30%	52.7 years
PCNL	1,637	9*	0.55%					60.6 years
Benign cystoscopic procedures	12,681	61	0.48%	26	0.21%	35	0.28%	52.3 years
Urethral dilatation procedures	12,900	30	0.23%	8	0.06%	22	0.17%	57.7 years
**Total**	**126,891**	**839**	**0.66%**	**373**	**0.29%**	**466**	**0.37%**	**65.2 years**

VTE = venous thromboembolism; DVT = deep vein thrombosis; PE = pulmonary embolism; TURBT = transurethral resection of bladder tumour; TURP = transurethral resection of prostate; PCNL = percutaneous nephrolithotomy

* absolute values not disclosed to preserve confidentiality

**Table 3:** Venous thromboembolism (VTE) incidence stratified by patient positioning

Position	Total	VTE	Incidence
Lithotomy	114,170	688	0.60
Supine	11,084	142	1.28*

* chi-squared test *p*<0.05

**Table 4:** Venous thromboembolism (VTE) incidence stratified by disease category for cystoscopic procedures

	Total	VTE	Incidence
Benign	78,405	407	0.52
Malignant	35,765	281	0.79*

* chi-squared test *p*<0.05

A systematic review of community-based epidemiological studies estimated the annual incidence of VTE to be around 0.1%.^9^ However, beyond the age of 40, there seems to be an exponential increase in VTE incidence, rising to 0.3–0.5% annually in patients aged between 70 and 79 years.

Unfortunately, there are inherent problems in using HES for the purposes of this study. It is not possible to control for of the use of thromboprophylaxis, pre-existing VTE risk factors (age, previous VTE, immobility, etc) or procedures performed under general or regional anaesthesia. Despite laparoscopic surgery being the established gold standard for a variety of procedures, HES do not account for different surgical techniques (open, laparoscopic or robotic). Furthermore, there is reliance on accurate clinical coding and also inevitable underestimation of VTE incidence given that some patients will be diagnosed with VTE out of the hospital setting.

Nevertheless, HES have been shown to be increasingly accurate and an effective tool in comparative audit.^10–12^ HES will no doubt continue to be used as a research tool in the future. The introduction of a greater number of codes for urological procedures performed with differing techniques would increase the usefulness of analogous analysis and is a recommendation of our study.

Despite its limitations, this study is the first large multi-centre study of postoperative VTE across a range of urological procedures. Comparing VTE incidence in our study with community-based control studies reveals a group of procedures that carry a VTE risk similar to and often less than the risk of developing a symptomatic VTE in the absence of any surgery. This calls into question the role for routine thromboprophylaxis in patients undergoing low-risk urological procedures.

The current guidance draws its conclusions from a number of single-centre randomised controlled trials conducted in the 1970s and 80s. The incidence of symptomatic VTE in those patients not receiving thromboprophylaxis was as high as 10% for DVT and 9% for PE following both transvesical and transurethral resection of prostate.^3–7^ We suggest that in the last 30 years there has been shift towards shorter operating times as well as earlier mobilisation and discharge from hospital. Despite increasing use of mechanical and medical thromboprophylaxis, these factors are highly likely to contribute to our study findings. The combination of dated evidence and apparently low VTE incidence in our study suggest value in conducting further randomised controlled trials to delineate the health benefit and economical implications of routine thromboprophylaxis, particularly for patients undergoing low-risk surgery.

## Conclusions

It would appear that there is scope for improvement and streamlining of care with regard to VTE prevention for patients undergoing both high-risk and low-risk urological surgery. Given the limitations of our study, alterations in local practice should await further investigation.
